# Mass vaccination with reassortment-impaired live H9N2 avian influenza vaccine

**DOI:** 10.1038/s41541-024-00923-y

**Published:** 2024-08-03

**Authors:** Flavio Cargnin Faccin, C. Joaquin Cáceres, L. Claire Gay, Brittany Seibert, Nick van Bentem, Luis A. Rodriguez, Ana Luiza Soares Fraiha, Matias Cardenas, Ginger Geiger, Lucia Ortiz, Silvia Carnaccini, Darrell R. Kapczynski, Daniela S. Rajao, Daniel R. Perez

**Affiliations:** 1 Department of Population Health, College of Veterinary Medicine, University of Georgia, Athens, GA USA; 2 Exotic and Emerging Avian Viral Disease Research Unit, Southeast Poultry Research Laboratory, U.S. National Poultry Research Center, Agricultural Research Service, USDA, Athens, GA USA; 3 Present Address: W. Harry Feinstone Department of Molecular Microbiology and Immunology, The Johns Hopkins Bloomberg School of Public Health, Baltimore, MD USA; 4 Present Address: Division of Farm Animal Health, Department Population Health Sciences, Faculty of Veterinary Medicine, Utrecht University, Utrecht, The Netherlands; 5 Present Address: Department of Microbiology, University of Texas Southwestern Medical Center, Dallas, TX USA; 6 Present Address: Departamento de Medicina Veterinária Preventiva, Universidade Federal de Minas Gerais, Belo, Horizonte, Minas Gerais Brazil; 7 Present Address: Department of Microbiology and Immunology, Emory University School of Medicine, Atlanta, GA USA

**Keywords:** Live attenuated vaccines, Influenza virus

## Abstract

Avian influenza poses a severe threat to poultry production and global food security, prompting the development of vaccination programs in numerous countries. Modified live virus (MLV) vaccines, with their potential for mass application, offer a distinct advantage over existing options. However, concerns surrounding reversion, recombination, and unintended transmission have hindered the progress of MLV development for avian influenza in poultry. To address these concerns, we engineered reassortment-impaired, non-transmissible, safe, immunogenic, and protective MLVs through the rearrangement of internal gene segments and additional modifications to the surface gene segments HA and NA. The unique peptide marker aspartic acid-arginine-proline-alanine-valine-isoleucine-alanine-asparragine (DRPAVIAN) was incorporated into HA, while NA was modified to encode the chicken interleukin-18 (ckIL18) gene (MLV-H9N2-IL). In vitro, the MLV-H9N2 and MLV-H9N2-IL candidates demonstrated stability and virus titers comparable to the wild-type H9N2 strain. In chickens, the MLV-H9N2 and MLV-H9N2-IL candidates did not transmit via direct contact. Co-infection studies with wild-type virus confirmed that the altered HA and NA segments exhibited fitness disadvantages and did not reassort. Vaccinated chickens showed no clinical signs upon vaccination, all seroconverted, and the inclusion of ckIL18 in the MLV-H9N2-IL vaccine enhanced neutralizing antibody production. A significant decrease in viral loads post-challenge underscored the protective effect of the MLVs. The MLV-H9N2-IL vaccine, administered via drinking water, proved immunogenic in chickens in a dose-dependent manner, generating protective levels of neutralizing antibodies upon aggressive homologous virus challenge. In summary, this study lays the groundwork for safe MLVs against avian influenza suitable for mass vaccination efforts.

## Introduction

The global poultry industry is economically and socially vital, supplying a cost-effective protein source. High-density conditions house approximately three-quarters of the world’s poultry, making birds susceptible to infectious diseases, notably avian influenza, responsible for substantial bird losses^1^. Avian influenza is caused by type A influenza viruses (FLUAVs) in the Orthomyxoviridae family, characterized by enveloped, pleomorphic particles with an 8-segment negative-sense RNA genome^2^. FLUAVs encode core viral proteins, including hemagglutinin (HA) and neuraminidase (NA), leading to subtypes based on their HA and NA antigenic properties^3^.

Wild aquatic birds are natural hosts for 17 HA and 9 NA subtype FLUAVs, allowing gene segment reassortment when co-infected with different FLUAVs^4,5^. FLUAVs are categorized as highly pathogenic avian influenza viruses (HPAIV) or low pathogenic avian influenza viruses (LPAIV), with H9N2 being a significant LPAIV in Galliformes, particularly prevalent in Asia, the Middle East, and Africa^6–8^. Recent H9N2 isolates exhibit a tri-basic cleavage site, enhancing transmission and replication in chickens^9^.

H9N2 infections in poultry result in delayed growth, lower egg production, and poor eggshell quality, often leading to secondary infections with respiratory pathogens and substantial economic losses^10–12^. The emergence of H9N2 strains has facilitated the reassortment of additional FLUAVs, including zoonotic strains such as H5Nx HPAIVs and Asian H7N9 LPAIV/HPAIV strains^13–15^. Poultry-adapted H9N2s have acquired zoonotic potential, binding to sialic acid receptors akin to human-adapted FLUAVs, prompting the World Health Organization to consider H9N2 LPAIVs as viruses of pandemic concern, with an increasing number of human cases^16–20^.

Vaccination programs against H9N2 in poultry have been implemented in Asia and the Middle East, but challenges include the magnitude of outbreaks, antigenic mismatch, and issues with vaccine potency^21–24^. Current vaccines, mainly whole-inactivated virus plus adjuvant (WIV-adj) and/or recombinant vector platforms, contribute to antigenic drift, necessitating individual administration and facing challenges related to prior immunity and vector issues^25–28^.

Modified live virus (MLV) vaccines, known for their success in eradicating diseases, can induce comprehensive immune responses, offering a potential mass vaccination solution for poultry^29,30^. Most successful vaccine strategies for poultry are based on MLV viruses such as Newcastle disease, Gumboro disease, Infectious laryngotracheitis, and Marek’s disease. MLV vaccines can potentially work in the presence of maternal antibodies^31^. In the case of avian influenza, concerns of reversion, recombination, and unintended transmission have slowed MLV development for use in poultry. We used reverse genetics to generate MLV vaccines against H9N2s based on genome rearrangement and incorporation of molecular markers on the HA and NA segments. These modifications led to MLVs with drastically reduced potential for reassortment and transmission. These MLV platforms were immunogenic and protective against H9N2 virus challenge. An immunomodulator was included in the MLV platform, MLV-H9N2-IL, the chicken interleukin-18 (ckIL18) that enhanced immune responses and protection in chickens after homologous virus challenge. MLV-H9N2-IL given via drinking water induced sterilizing immunity in a vaccine dose-dependent manner in chickens. These studies show the potential of utilizing MLVs for mass vaccination strategies, a highly desired attribute for implementation by the poultry industry. An argument can be made that a successful vaccine/vaccination strategy against H9N2 LPAIV is likely going to drastically reduce the emergence of other FLUAV subtypes of animal and/or public health concerns.

## Results

### The MLV-H9N2 and MLV-H9N2-IL vaccine candidates, featuring rearranged genomes and modifications in HA and NA segments, exhibit stability after serial passage in vitro

The viruses were developed based on the prototypical WF10 H9N2 strain with a rearranged genome (RAM), involving the placement of the M2 open reading frame (ORF) downstream of PB1 and the introduction of stop codons in the original M2 (Supplementary Fig. 1). In the RAM backbone, M2 is predicted to be co-translationally separated from PB1 via the action of the Thosea asigna 2 A protease (2ATav) sequence encoded between the PB1 and M2 ORFs. The MLV-H9N2 and MLV-H9N2-IL candidates carry a segment 4 (HA) encoding a 58 amino acid long sequence containing the unique DRPAVIAN peptide sequence placed downstream of the HA signal peptide sequence but upstream of the mature HA ORF (Supplementary Fig. 1). The modified HA segment was derived from the sequence of a chicken H9N2 isolate identified in Egypt in 2018 (ck/EGY). The DRPAVIAN peptide is predicted to be co-translationally removed from the HA allowing bona fide expression of HA trimers on the virus surface. The DRPAVIAN peptide sequence is unique to the MLV-H9N2 and MLV-H9N2-IL candidates and does not exist in any known sequence of any living organism available in public databases. The unique sequence is expected to reduce the HA fitness for reassortment and can be used as a differentiation method compared to wild-type HA segments.

**Fig. 1:**
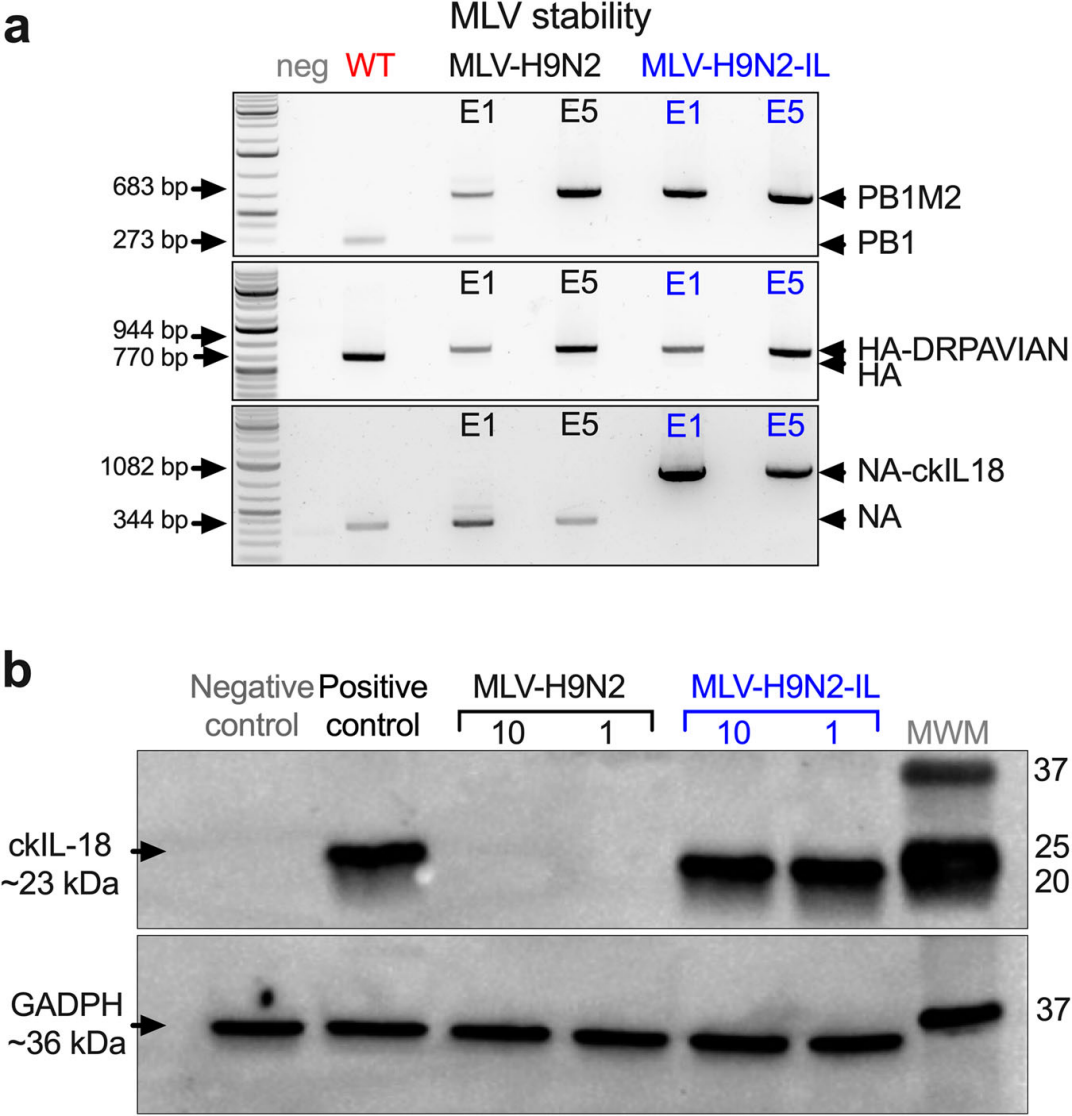
The MLV-H9N2 and MLV-H9N2-IL vaccine candidates exhibit stability. **a** RT-PCR fragment profiles of MLVs before serial passages in eggs (E1) or after serial passages in eggs (E5) targeting the PB1-M2 (top), HA-DRPAVIAN (middle), and NA-IL-18 (bottom). Wild-type segments were included as positive controls to demonstrate the differences in size between modified and non-modified segments. **b** Chicken IL-18 expression from the MLV-H9N2-IL virus by western blot. Protein lysates of MDCK cells inoculated with MLV-H9N2 (black) or MLV-H9N2-IL (blue) at an MOI of 10 or 1 as indicated. Positive control corresponds to protein lysates of HEK293T cells transfected with the control expression plasmid pCAGGS expressing N2WF10-Furin-2A-IL-18-HIS. Negative control corresponds to non-transfected HEK293T cell lysates. The arrows indicate the predicted molecular weight of chicken IL-18 (~23 KDa) and the host cellular protein glyceraldehyde-3-phosphate dehydrogenase (GAPDH; ~36 KDa) used as a gel loading control. Molecular weight marker (MWM) with numbers on the right in KDa of marker protein bands. Western blot images derived from the same set of protein lysates. Uncropped figures are shown in Supplementary Fig. 3.

In the MLV-H9N2-IL candidate, the ckIL18 sequence was added to the C-terminus of the N2 NA, separated by a spacer released during translation via 2ATav activity (Supplementary Fig. 1). The MLV-H9N2 and MLV-H9N2-IL virus vaccine candidates were initially rescued in embryonated SPF chicken eggs and labeled as egg passage 1 (E1). The E1 MLV-H9N2 and MLV-H9N2-IL virus stocks were serially passaged five times in eggs (E5) to monitor the stability of genome modifications. RT-PCRs were performed targeting each modified segment from the E1 and E5 virus passages. The sizes of the PCR products were maintained in both E1 and E5 passages which were bigger than those obtained from the unmodified segment controls (Fig. 1a). Genome stability was further confirmed by Sanger sequencing with no discernible mutations in any of the modified segments.

To demonstrate whether genome rearrangements affect virus growth, MDCK cells were infected at an MOI of 0.01 and incubated at 37 °C or 41 °C for 96 h. Compared with the H9N2 WT virus, the MLV-H9N2 and MLV-H9N2-IL viruses showed similar virus replication at 37 °C, with minor differences at 12 (hours post-infection) hpi but similar replication levels at 24, 48, 72, and 96 hpi (Supplementary Fig. 2a). At 41 °C, both MLVs showed similar virus replication compared to the H9N2 WT virus (Supplementary Fig. 2b). In contrast, the control H9N2att showed impaired replication at 41 °C but not 37 °C due to the introduction of temperature-sensitive mutations^29^. To further evaluate the replication of the MLVs, growth kinetics were performed in human-derived cells. Specifically, human lung carcinoma (A549) cells and immortalized human airway epithelial cells (HAE-BCi-NS1.1^32^) grown in an air-liquid interface were infected at an MOI of 0.01 and incubated at 37 °C or 41 °C for 96 h. In A549 cells, the MLV-H9N2-IL replicated significantly less than a prototypic pandemic H1N1 virus^33^ (pH1N1) at all timepoints at 37 °C, demonstrating an attenuated profile (Supplementary Fig. 2c). Similar results were observed with the MLV-H9N2. Of note, both MLVs replicated less than the WT H9N2 virus. Interestingly, no statistically significant differences were observed in virus replication between the MLVs and the pH1N1 at 41 °C, but all viruses showed limited virus replication in human cells at this temperature (Supplementary Fig. 2d). Both MLV-H9N2-IL and MLV-H9N2 viruses replicated significantly less than the pH1N1 virus in HAE cells at 37 °C and 41 °C (Supplementary Fig. 2e, f), with replication levels below 3 log_10_ TCID_50_eq/ml. These MLVs also replicated less than the WT H9N2 at both temperatures. Overall, our data showed that the MLV-H9N2 and MLV-H9N2-IL are attenuated in human cell lines, exhibiting significantly lower replication levels compared to a pandemic H1N1 virus.

The E4 passage virus was used to prepare an E5 stock which was used for vaccine studies. The E5 stock of the MLV-H9N2 reached titers of 3.16 × 10^8^ EID_50_/ml while the MLV-H9N2-IL reached titers of 6.81 × 10^7^ EID_50_/ml. Overall, these studies showed that the MLV-H9N2 and MLV-H9N2-IL viruses are stable and grow to high titers, characteristics that make them potentially suitable vaccine candidates. To demonstrate that IL-18 is being expressed from the MLV-H9N2-IL virus, MDCK cells were inoculated with the MLV-H9N2-IL and MLV-H9N2 viruses at low (1) and high (10) MOI. Cell lysates from HEK293T cells transfected with a plasmid expressing the N2WF10-Furin-2A-IL-18 product were used as positive control. IL-18 expression was detected from the MLV-H9N2-IL virus, regardless of the MOI employed (Fig. 1b and Supplementary Fig. 3a). As expected, we did not see IL-18 expression from the control MLV-H9N2 virus.

### Modifications made to the HA and NA segments affect their fitness for reassortment both in vitro and in vivo

**Fig. 2:**
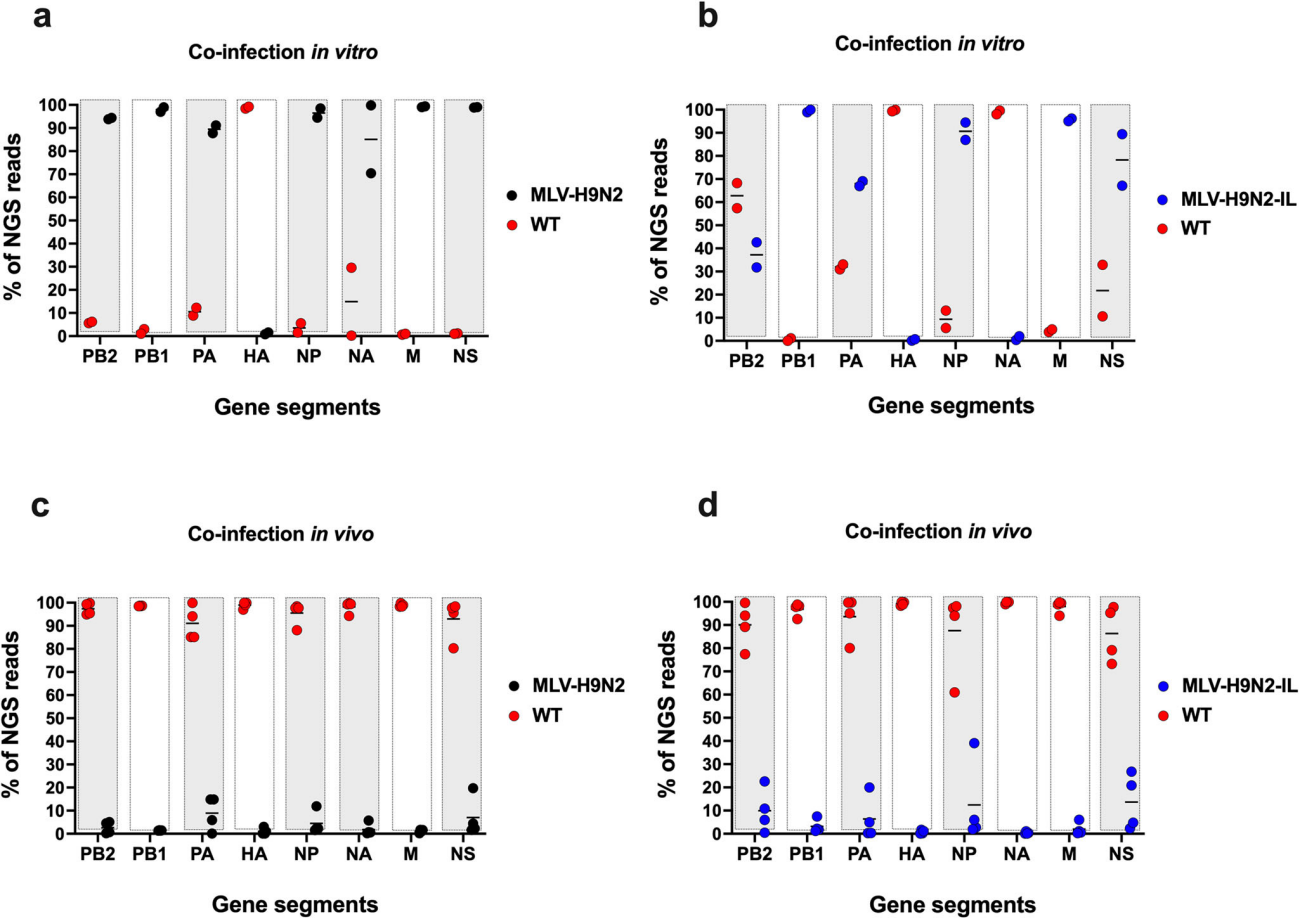
Reassortment data upon co-infections in vitro and in vivo. MDCK cells were infected with H9N2 WT (WT) virus and either **(a**) MLV-H9N2 or (**b**) MLV-H9N2-IL viruses. MDCK cells were coinfected with a 10:1 MLV/WT virus ratio. Supernatants collected at 72 hpi underwent two rounds of limiting dilution followed by NGS sequencing. FASTQ files were used to calculate the proportion of NGS reads that matched MLV or WT for each specific segment. Reassortment data upon co-infection of 2-week-old chickens with H9N2 WT virus and either (**c**) MLV-H9N2 or (**d**) MLV-H9N2-IL viruses at a 10MLV:1WT ratio. OP swabs collected at 5 dpi were sequenced by NGS and analyzed as the in vitro data. Data is graphed as % proportion of reads versus gene segment. WT segments (red), MLV-H9N2 segments (black), MLV-H9N2-IL segments (blue) in the progeny viruses are shown. Gray shading rectangles represent unmodified viral gene segments in WT and MLV strains. White shading rectangles represent modified viral gene segments in the MLV strains but unmodified viral gene segments in WT.

Co-infection studies in MDCK cells were conducted to assess the reassortment potential of the modified segments in the vaccine virus. These studies involved co-infecting cells with either the MLV-H9N2 or MLV-H9N2-IL strain and a prototypic WT H9N2 strain (ck/Tun) at a ratio of 10:1 (MLV:WT). Following two rounds of virus cloning by limiting dilution, next-generation sequencing (NGS) data revealed that the DRPAVIAN modification rendered the modified HA less fit than the WT HA from the ck/Tun strain. None of the progeny virus clones contained the DRPAVIAN-modified HA segment (Fig. 2a, b). Similarly, the ckIL18-modified NA was largely outcompeted by the WT NA from the ck/Tun strain during co-infection in vitro (Fig. 2b). In contrast, the modified PB1-M2 and M1∆M2 segments did not exhibit a significant fitness disadvantage and behaved similarly to unmodified gene segments (Fig. 2a, b). These in vitro studies indicated that the modifications introduced into the HA and NA segments, but not those in PB1 and M, affected segment fitness.

To further evaluate reassortment potential in vivo, co-infection studies were conducted in two-week-old chickens using a 10:1 (MLV:WT) ratio to favor reassortment. Oropharyngeal (OP) swabs collected on day 5 post-inoculation (dpi) were sequenced using NGS. Consistent with the in vitro findings, both MLV-H9N2 and MLV-H9N2-IL exhibited a clear fitness disadvantage compared to the WT virus in vivo and were outcompeted (Fig. 2c, d). Importantly, reassortment between the MLV viruses and the WT virus was not observed in vivo. Interestingly, the modified PB1-M2 and M1∆M2 segments, which did not exhibit a significant fitness disadvantage in vitro, demonstrated reduced fitness in vivo, along with all other gene segments. Collectively, these data demonstrate that the MLVs are safe and possess a fitness disadvantage compared to the WT virus.

### MLV-H9N2 and MLV-H9N2-IL are not transmissible to direct contact chickens

**Fig. 3:**
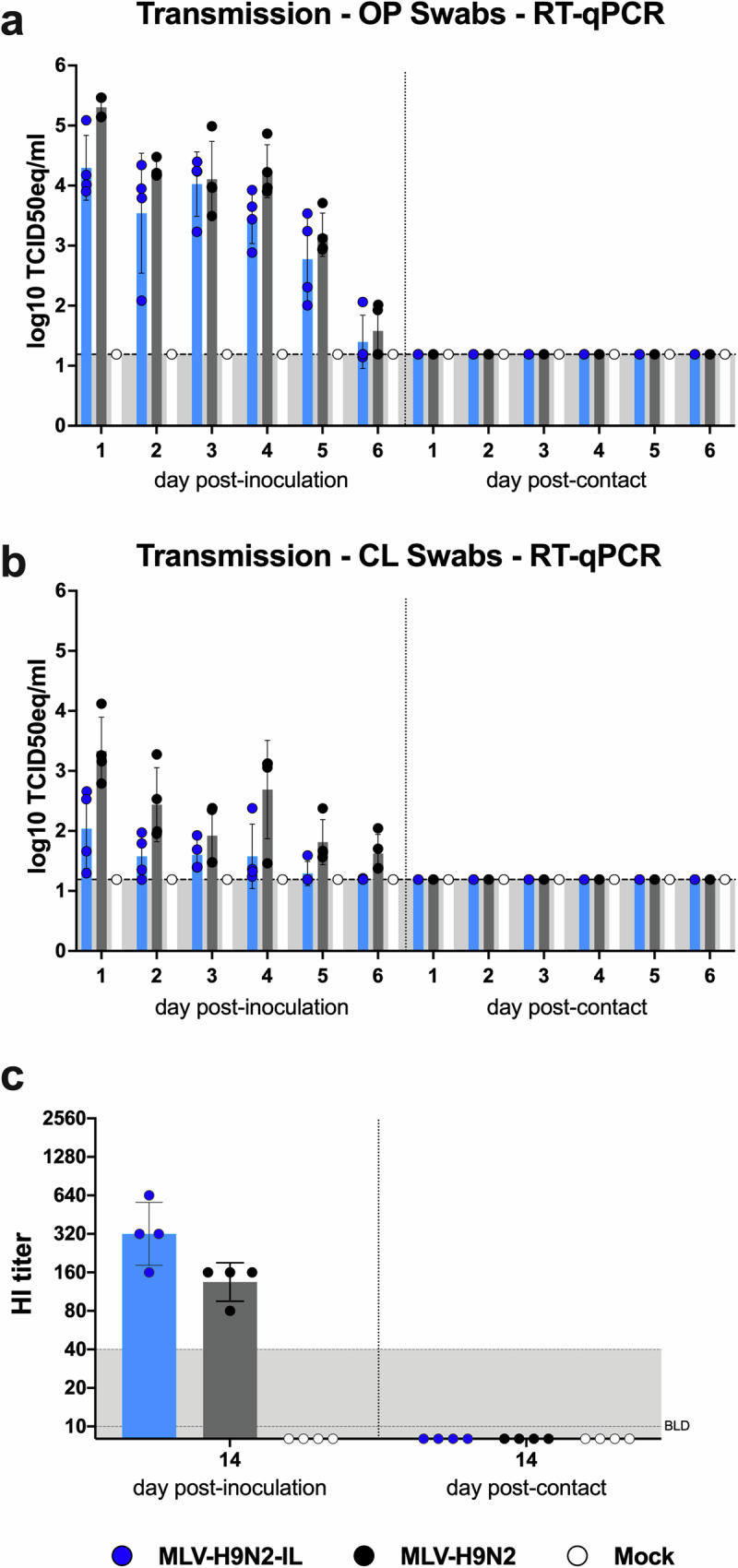
MLVs transmissibility in 2-weeks-old chickens. Animals (*n* = 4/group) were directly inoculated with either the MLV-H9N2 (black) or MLV-H9N2-IL (blue) strains at 1 × 10^6^ EID_50_/chicken. Naïve direct contacts were added 24 hpi. **a** OP swabs and (**b**) CL swabs were collected (*n* = 4/group/day) every day from 1 to 6 days after inoculation or after contact to analyze transmission between directly inoculated and direct contact animals. Viral loads by RT-qPCR shown as the mean ± SD Log_10_ TCID_50_ equivalent/mL, LOD 1.199 Log_10_ TCID_50_ equivalent/mL. **c** Blood samples were collected (*n* = 4/group) at 14 dpi and 14 dpc and sera prepared for HI assays to analyze seroconversion using 2-fold serum dilutions.

To evaluate the potential for transmission of MLV-H9N2 and MLV-H9N2-IL viruses in vivo in chickens, their ability to transmit to naïve direct contact chickens was assessed. Two-week-old chickens were inoculated with either MLV-H9N2 or MLV-H9N2-IL virus via the eyes and the respiratory (nasal and tracheal), oral, and cloacal routes (EROC) at a dose of 1 × 10^6^ EID_50_/chicken. Naïve direct contact chickens were introduced 24 hpi, and OP and cloacal (CL) swabs were collected from both inoculated and contact chickens daily. Viral RNA was detected in OP and CL swabs collected from directly inoculated chickens from 1 to 6 dpi, with a trend towards lower viral RNA (vRNA) levels in chickens inoculated with MLV-H9N2-IL compared to those inoculated with MLV-H9N2. Notably, none of the direct contact chickens exhibited detectable levels of vRNA in either OP or CL swabs at any timepoint (Fig. 3a,b). Additionally, serological analysis revealed no seroconversion in direct contact chickens at 14 days post-contact (Fig. 3c). These findings suggest that the replication levels of the MLVs in vivo are insufficient to allow for transmission to naïve direct contact birds.

### MLVs generate serum-neutralizing antibodies predictive of protection

**Fig. 4:**
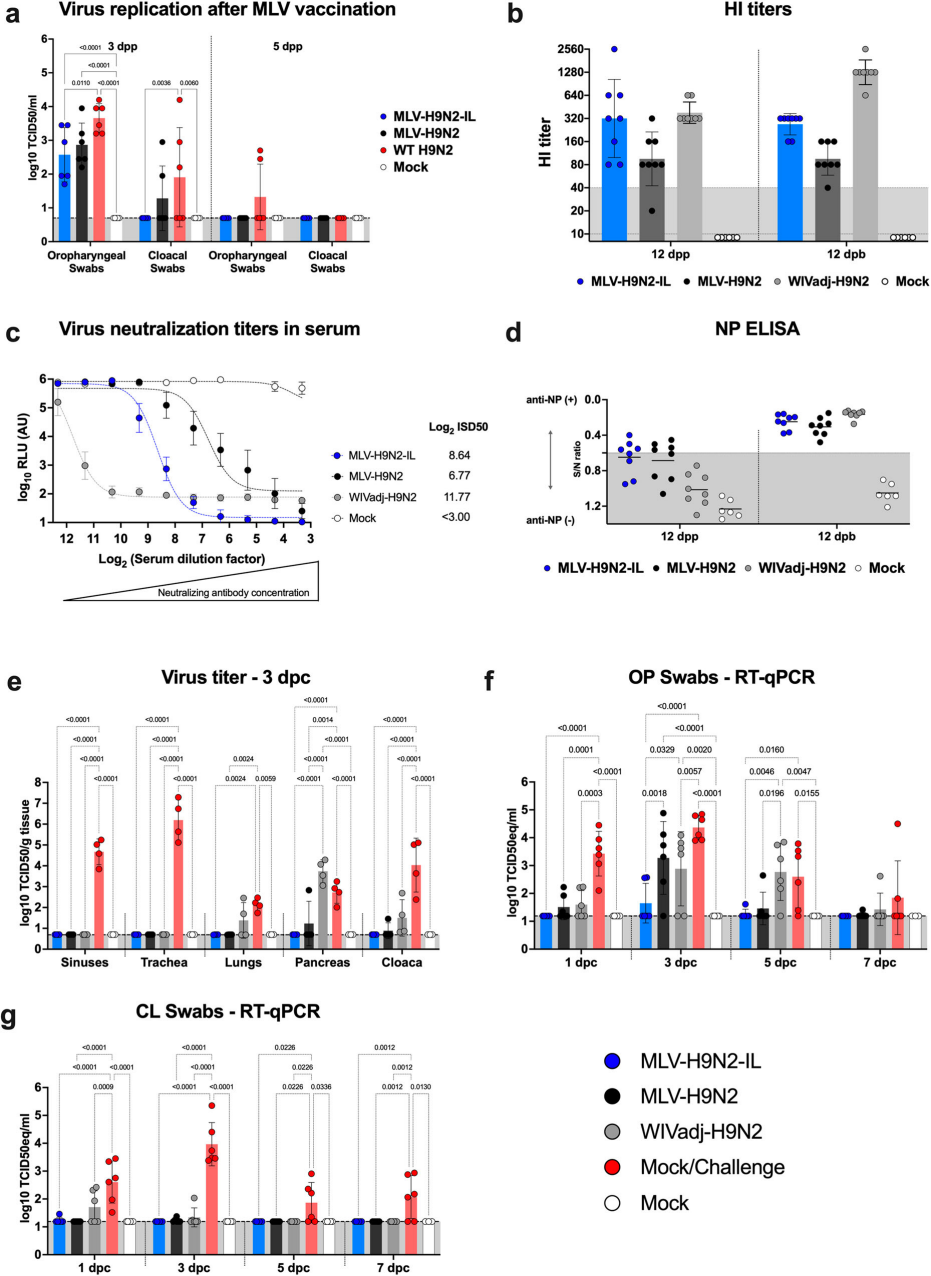
MLVs are safe, generate a strong immune response, and protect chickens upon homologous challenge. **a** After virus inoculation, OP and CL swabs were collected (*n* = 6/group) at 3- and 5-dpp to establish levels of virus shedding shown as the mean ± SD Log_10_TCID_50_/mL, LOD 0.699 Log_10_ TCID_50_/mL. Virus shedding for MLV-H9N2-IL (blue), MLV-H9N2 (black), H9N2 WT virus (red) and mock inoculated controls (white) are shown. Note that the MLV-H9N2-IL (blue) replicates significantly less than the H9N2 WT virus (red). **b**–**d** Blood samples were collected (*n* = 8/group/timepoint) at 12 dpp and 12 dpb and sera prepared to establish levels of seroconversion from the MLV-H9N2-IL (blue), MLV-H9N2 (black), WIVadj-H9N2 (gray), and mock-vaccinated controls (mock). **b** HI titers expressed as 2-fold serum dilution versus timepoint of serum collection. **c** VNluc titers plotted as arbitrary Log_10_ relative light units (Log_10_ RLU (AU)) versus the Log_2_ sera dilution. Nluc activity was measured at 48 hpi. The Log_2_ inhibitory sera dilution 50 (Log_2_ ISD50) for each serum is shown. **d** Levels of NP antibodies as measured by a commercial ELISA and expressed as Signal-to-Noise (S/N) ratio versus timepoint of serum collection. S/N ≥ 0.6 is considered positive (**e**) Sinuses, trachea, lungs, pancreas, and cloaca samples (*n* = 4/group) were collected at 3 dpc and subjected to virus titration and shown as the mean ± SD Log_10_ TCID_50_/mL**. f** OP swabs and (**g**) CL swabs were collected (*n* = 6/group/day) every other day from 1 to 7 dpc. Virus shedding measured by RT-qPCR and expressed as the mean ± SD Log_10_ TCID_50_ equivalent/mL versus day post-challenge with LOD 1.199 Log_10_ TCID_50_ equivalent/mL. Data analysis and graphs were prepared using Prism v10 (GraphPad). Ordinary two-way ANOVA was performed to calculate *P* values followed by Tukey’s multiple comparison tests. Only statistically significant differences are shown.

To evaluate the immunogenicity of MLV-H9N2 and MLV-H9N2-IL viruses, we employed a prime-boost vaccination strategy administered two weeks apart. Two-week-old chickens were inoculated via the EROC route with a dose of 1 × 10^6^ EID_50_/chicken of either MLV-H9N2 or MLV-H9N2-IL. A control group received a subcutaneous (SC) injection of a WIVadj-H9N2 vaccine prepared in-house at a dose of 512 hemagglutinin units (HAU) per dose. A fourth group of chickens served as mock controls and were inoculated with PBS. OP and CL swabs were collected after prime vaccination to assess virus replication levels. Both MLV strains exhibited a limited window of replication, with detectable virus in both OP and CL swabs on day 3 (Fig. 4a). Notably, MLV-H9N2-IL loads were significantly less than the H9N2 WT control in OP swabs on day 3, consistent with the reduced levels of vRNA observed in the previous study (Fig. 3a, b). No MLV virus was detected after boost vaccination (data not shown).

Serum samples collected at 12 days post-prime (dpp) and 12 days post-boost (dpb) revealed the presence of measurable HI and virus neutralization titers (VNluc), as well as anti-NP antibodies, in all vaccinated groups except for the PBS control group. The WIVadj-H9N2 vaccine group generated the highest HI titers after prime (mean titer of 320) and after boost (mean titer of 1280). Interestingly, the inclusion of IL-18 in the MLV-H9N2-IL vaccine resulted in higher HI titers after prime (mean titer of 320 versus 80) and after boost (mean titer of 320 versus 80) compared to the vaccine candidate without IL-18 (MLV-H9N2) (Fig. 4b). To assess VNluc titers, we employed a recombinant H9N1 homologous influenza virus expressing Nanoluciferase, where titers are inversely proportional to its activity. Consistent with the HI assay, the WIVadj-H9N2 vaccine induced the highest levels of neutralizing antibodies (Log_2_ ISD_50_ of 11.77), and MLV-H9N2-IL elicited higher levels of neutralizing antibodies (Log_2_ ISD_50_ of 8.64) compared to MLV-H9N2 (Log_2_ ISD_50_ of 6.77) (Fig. 4c), suggesting that IL-18 may play a role in enhancing the host immune response following vaccination and inducing higher levels of neutralizing antibodies indicative of protection. Both MLVs induced higher levels of anti-NP antibodies after prime vaccination compared to the WIVadj-H9N2 vaccine (Fig. 4d), although all means were below the threshold for positive samples (0.6). After boost vaccination, anti-NP antibody levels increased in all groups and were comparable between the MLVs and the WIVadj-H9N2 vaccine. In conclusion, our findings demonstrate that MLVs are attenuated in vivo and induce neutralizing antibodies predictive of protection following vaccination.

### MLVs, but not the WIVadj-H9N2 vaccine, significantly reduce viral loads and protect chickens after a homologous challenge

To evaluate the protective efficacy of MLVs and the WIVadj-H9N2 vaccine, two weeks post-boost vaccination, chickens were challenged with a homologous virus at a dose of 1 × 10^8^ EID_50_/chicken via the EROC route. CL and OP swabs were collected every other day until 7 days post-challenge (dpc), and tissues were collected at 3 dpc.

In the sinuses, trachea, and cloaca, all vaccines significantly reduced viral loads compared to the mock-vaccinated challenge (Fig. 4e). In the lungs, viral replication levels were similar between the WIVadj-H9N2 vaccine and mock challenge. Interestingly, viral replication levels in the pancreas were higher in the WIVadj-H9N2 vaccine group compared to the mock challenge, although the difference was not statistically significant. Both MLVs effectively reduced viral loads and completely protected the birds against the homologous virus in all tissues examined (Fig. 4e). In OP swabs, the MLV-H9N2-IL vaccine led to significant reduction in virus shedding. At 3 dpc, the inclusion of IL-18 in the MLV-H9N2-IL vaccine significantly reduced viral loads and enhanced protection compared to the vaccine candidate without IL-18 (Fig. 4f). Importantly, in OP swabs, the WIVadj-H9N2 vaccine did not reduce virus shedding at 3 and 5 dpc compared to the mock challenge (Fig. 4f). In CL swabs, all vaccines significantly reduced viral loads and fully protected the birds against a homologous challenge compared to the mock challenge (Fig. 4g). Overall, our findings demonstrate that MLVs provide superior protection against a homologous challenge compared to the WIVadj-H9N2 vaccine.

### MLV-H9N2-IL is stable, shows limited replication, and is immunogenic during vaccine administration via drinking water

**Fig. 5:**
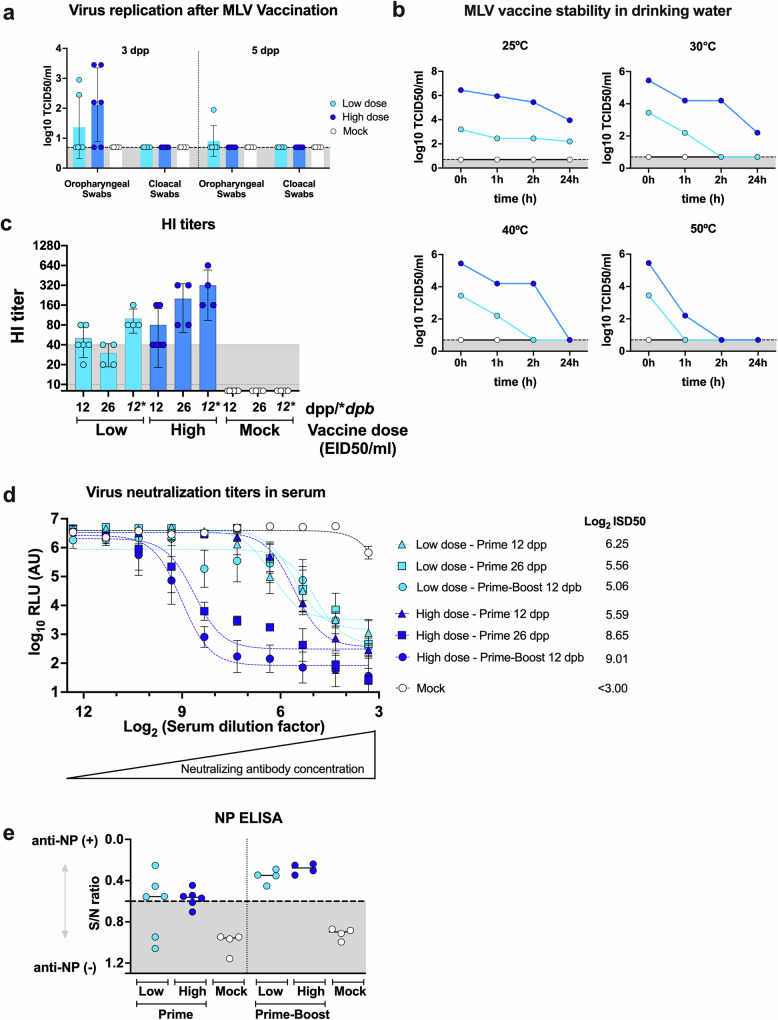
MLV-H9N2-IL is stable and immunogenic when given via drinking water. **a** Chickens were vaccinated with MLV-H9N2-IL via drinking water for 2 h. Low dose: 10^4^ EID_50_/mL (light blue). High dose: 10^6^ EID_50_/mL (dark blue). Mock vaccinated controls included (white). OP and CL swabs were collected (*n* = 6/group) at 3- and 5- dpp. **b** MLV-H9N2-IL vaccine stability in drinking water analyzed by incubating the two vaccine doses at 25 °C, 30 °C, 40 °C, and 50 °C. Timepoints were collected at 0 h, 1 h, 2 h, and 24 h. Virus in samples were then titrated and shown as the mean ± SD Log_10_ TCID_50_/mL, LOD 0.699 Log_10_ TCID_50_/mL. Sera prepared from blood samples collected at 12 dpp (*n* = 6/group), 26 dpp (*n* = 4/group), and 12 dpb (*n* = 4/group) from the low dose (light blue) and high dose (dark blue) MLV-H9N2-IL vaccine groups were used to establish seroconversion. **c** HI titers (**d**), VNluc titers, and (**e**) NP antibody titers were established as detailed in Fig. 4.

To assess the feasibility of mass vaccination using the most promising vaccine candidate, MLV-H9N2-IL, two-week-old chickens were vaccinated via drinking water for 2 h with either 1 × 10^4^ EID_50_/ml (low dose) or 1 × 10^6^ EID_50_/ml (high dose) of MLV-H9N2-IL, employing either a prime-only or prime-boost strategy. OP and CL swabs were collected to evaluate virus replication levels following drinking water vaccination. MLV-H9N2-IL exhibited dose-dependent and limited virus replication in OP swabs on day 3 post-prime (Fig. 5a). Infectious virus was not detected in CL swabs (Fig. 5a).

To assess vaccine stability in drinking water, vaccine samples were mixed with distilled water and incubated at various temperatures (25 °C, 30 °C, 40 °C, and 50 °C) for 2 h. Chickens were allowed to drink water for 2 h, and during this period, MLV-H9N2-IL was found to be stable at both low and high dose at 25 °C (Fig. 5b). Additionally, at 30 °C and 40 °C, a high dose remained stable for 2 h, while low dose appeared to decrease, with no infectious virus detected after 2 h at these temperatures. Finally, titers rapidly declined when samples were incubated at 50 °C (Fig. 5b).

Blood samples were collected at 12 dpp and 12 dpb to measure HI titers, neutralizing antibodies, and anti-NP antibodies. For the prime-only groups, blood was also collected at 26 dpp, two days prior to the challenge. In the low dose group, HI titers increased from around 40 (20–80) after prime to 80 (80–160) following the boost (Fig. 5c). Similarly, HI titers increased in the high dose group after the boost (average 320, 160-640) compared to prime (average 80, 40–160). Notably, HI titers increased from 80 to 160 when comparing 12 dpp to 26 dpp in the high dose group.

Consistent with the HI data, VNluc assays revealed similar and consistent results, with a prime-only (Log_2_ ISD_50_ of 8.65) or prime-boost (Log_2_ ISD_50_ of 9.01) regimen using high dose generating the highest levels of neutralizing antibodies (Fig. 5d). Anti-NP antibodies were also detected after prime vaccination and increased after boost, with comparable levels observed between the two doses tested (Fig. 5e). In summary, these findings demonstrate that MLV-H9N2-IL is stable during vaccine administration, remains attenuated when administered via drinking water, and induces neutralizing antibodies predictive of protection following vaccination.

### Prime-Boost with 10^6^ EID_50_/ml of MLV-H9N2-IL induces sterilizing immunity

Two weeks after the boost vaccination or four weeks after the prime vaccination, chickens were challenged with a homologous virus at a dose of 1 × 10^8^ EID_50_/chicken via the EROC route. OP and CL swabs were collected every other day until 5 dpc, and tissues were collected at 3 dpc.

**Fig. 6:**
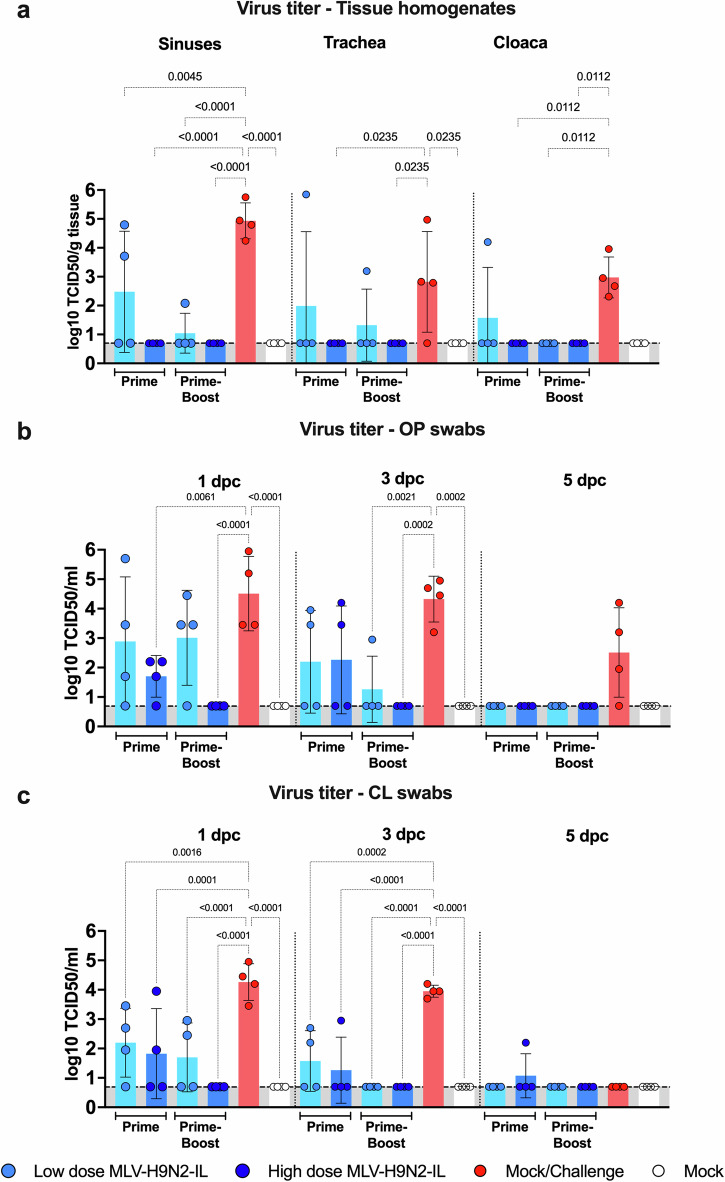
Prime-Boost with 10^6^ EID_50_/ml of MLV-H9N2-IL induces sterilizing immunity. **a** Sinuses, trachea, and cloaca samples (*n* = 4/group) were collected at 3 dpc and viruses titers established as the mean ± SD Log_10_ TCID_50_/gr of tissue homogenate, LOD 0.699 Log_10_ TCID_50_/gr. **b** OP swabs and (**c**) and CL swabs were collected (*n* = 4/group/day) every other day from 1 to 5 dpc. Virus titers shown as the mean ± SD Log_10_ TCID_50_/mL, LOD 0.699 Log_10_ TCID_50_/mL. Data analysis and graphs performed with Prism v10 (GraphPad) using ordinary two-way ANOVA, *P* values calculated by Tukey’s multiple comparison tests. Only statistically significant differences are shown.

Tissue analysis revealed that all doses and vaccination strategies protected against the homologous challenge by reducing viral loads in the sinuses (Fig. 6a). Similar results were observed in the cloaca (Fig. 6a). In the trachea, a decrease in viral loads was observed compared to the mock challenge, but the difference was not statistically significant.

In OP swabs, prime-only and prime-boost vaccination with high dose significantly reduced viral loads compared to the mock challenge at 1 dpc (Fig. 6b). Prime-only or prime-boost vaccination with low dose also decreased viral loads but lacked statistical significance. On 3 dpc, a boost vaccination was required to significantly reduce viral loads compared to the mock challenge, regardless of the dose (Fig. 6b). All doses and strategies significantly reduced viral loads at 5 dpc. Importantly, prime-boost vaccination with high dose completely prevented virus shedding in OP swabs on days 1, 3, and 5 dpc (Fig. 6b).

In CL swabs, all vaccine doses, and strategies (low dose prime-only, high dose prime-only, low dose prime-boost, and high dose prime-boost) significantly reduced viral loads and protected the birds against the homologous virus at 1 and 3 dpc (Fig. 6c). Notably, vaccination with a prime-boost regimen at high dose completely prevented virus shedding in CL swabs after the challenge.

In conclusion, prime-boost vaccination with high dose of MLV-H9N2-IL via drinking water induces sterilizing immunity in chickens, as evidenced by the absence of detectable virus replication in OP swabs, CL swabs, and the sinuses, trachea, and cloaca.

## Discussion

The majority of currently approved and widely used avian influenza vaccination programs worldwide, including those in Mexico, Egypt, Pakistan, and China, rely primarily on adjuvanted inactivated whole virus vaccines^34^. It is well recognized that inactivated vaccines induce robust serum antibody responses but fail to elicit effective mucosal and cell-mediated immunity^35^. Moreover, they are incapable of activating pattern-recognition receptors on infected cells^36^, which play a crucial role in enhancing the production of pro-inflammatory cytokines and type I interferons, thereby initiating the local cellular innate immune response^37^. Another significant drawback is the potential for mismatches between circulating and vaccine strains, allowing the virus to evolve and acquire mutations that could increase its replication and transmission capabilities^38^. Studies have demonstrated that these vaccines fail to reduce virus shedding and do not prevent the transmission of H9N2 in chickens^27^, highlighting their inability to disrupt the influenza transmission cycle.

MLVs hold promise for developing broader and longer-lasting effective vaccines against avian influenza due to their ability to replicate and mimic natural infection^35,39^. Our laboratory has successfully demonstrated that genome rearrangement is a viable, safe, and efficacious approach for MLV development^40–42^. In previous studies, our RAM vaccines effectively protected mice against clinical disease and mortality^40^. Additionally, the incorporation of a natural adjuvant into the HA segment enhanced the stimulation of IgG and IgA responses^41^.

Building upon these findings, we developed two MLVs, MLV-H9N2 and MLV-H9N2-IL, based on genome rearrangement of the H9N2 subtype, the most prevalent LPAIV circulating in chickens worldwide. Utilizing the previously established RAM approach as an attenuated backbone, we incorporated additional molecular markers into the HA to prevent reassortment and an immunomodulator into the NA to further stimulate protective antibody responses.

To assess the genetic stability of MLV-H9N2 and MLV-H9N2-IL viruses, we subjected them to five serial passages in eggs and evaluated their genetic integrity using RT-PCR and Sanger sequencing. The results confirmed that both viruses maintained their genetic integrity throughout the serial passages.

Interestingly, both the DRPAVIAN modification and the inclusion of IL-18 in the NA resulted in a fitness disadvantage compared to the wild-type (WT) segments from the ck/Tunisia strain in vitro. In contrast, the modified PB1-M2 and M1∆M2 segments were detected in the progeny virus and exhibited similar behavior to unmodified segments in vitro. One possible explanation for the presence of these modified MLV segments in the progeny virus is the fact that MLVs were not attenuated in vitro. Despite several gene modifications, both MLVs replicated to similar levels as an H9N2 WT virus at 37 °C and 41 °C in MDCK cells. Furthermore, we also demonstrated that the MLV-H9N2 and MLV-H9N2-IL replicate significantly less than a pandemic H1N1 virus in human-derived cells. In vivo, both MLVs exhibited an attenuated profile, with virus replication levels not exceeding 3 log_10_ TCID_50_/ml and restricted to within 3 to 5 days post-prime. These low levels of virus replication in directly inoculated animals prevented the transmission of MLV-H9N2 and MLV-H9N2-IL to naïve direct contact chickens. Consistent with the attenuated profile of MLVs in vivo, our in vivo co-infection data demonstrated that both MLVs have a fitness disadvantage and all MLV segments were outcompeted by the WT virus in vivo. Additionally, we did not observe any reassortment between our MLV viruses and the WT virus in vivo. Notably, the modified PB1-M2 and M1∆M2 segments exhibited reduced fitness in vivo, further corroborating their safe profile. The observed discrepancy between in vitro and in vivo replication highlights a desirable characteristic of live attenuated vaccines. Efficient replication in cell cultures or embryonated eggs is essential for large-scale vaccine production. Conversely, attenuation in the target host is crucial for safety and efficacy. Our MLVs exhibit both of these attributes, making them promising candidates for vaccine development.

However, we must exercise caution when interpreting these data, as the MLVs produced in this study contain five (MLV-H9N2) and four (MLV-H9N2-IL) WT segments that could potentially be freely exchanged. Although it was not the case, we anticipated that unmodified MLV segments (PB2, PA, NP, NS) may exhibit a fitness advantage compared to the WT ck/Tun strain, as these are also WT segments from the WF10 virus and can potentially outcompete the ck/Tun strain. Further investigations are warranted to determine whether additional modifications to each gene segment could be tolerated without compromising virus yield and protective efficacy while further minimizing reassortment potential. Such studies, however, are beyond the scope of this report.

We further investigated the humoral immune responses elicited by vaccination via the EROC route. As anticipated, the WIVadj-H9N2 vaccine induced the highest levels of HI titers and neutralizing antibodies. While both MLVs generated lower HI titers, their responses were consistent with antibody levels considered protective. This aligns with our previous studies using alternative MLVs against type A and B influenza viruses and contrasts with the limited seroconversion typically associated with currently approved cold-adapted seasonal influenza vaccines for human use^40,41^.

Despite the robust humoral responses in the WIVadj-H9N2 vaccine group, these were not sufficient to fully protect the birds against the homologous H9N2 challenge. We still detected virus in the lungs and OP swabs of birds immunized with the WIVadj-H9N2 vaccine, confirming the inability of inactivated vaccines to reduce viral loads in vaccinated birds. Notably, chickens vaccinated with the WIVadj-H9N2 vaccine exhibited significant virus replication after challenge, in samples collected from the pancreas. This unexpected finding may partially explain the ineffectiveness of inactivated vaccines in the field against H9N2 LPAIVs. Conversely, MLV-vaccinated birds, especially those receiving the MLV-H9N2-IL vaccine, exhibited almost complete protection against the challenge. This highlights the superior efficacy of MLVs in contrast to inactivated vaccines.

IL-18, a proinflammatory cytokine belonging to the interleukin-1 family, was initially identified as an interferon-gamma-inducing factor^43^. IL-18 plays a crucial role in the host’s response to influenza A virus. Studies involving chickens immunized with a recombinant fowl pox virus expressing H9 HA and chicken IL-18 suggest that IL-18 can enhance anti-HA responses when co-expressed^44^. An eukaryotic expression plasmid encoding chicken IL-18 has been employed as a potential adjuvant in conjunction with an inactivated Newcastle disease vaccine, resulting in elevated antibody level responses and T and B lymphocyte proliferation compared to the group that received only the inactivated vaccine^45^.

Consistent with these findings, our studies demonstrated that the inclusion of IL-18 elicited higher levels of HI titers and neutralizing antibodies compared to the same candidate without IL-18 (MLV-H9N2). Additionally, we observed enhanced protection in chickens vaccinated with MLV-H9N2-IL, as evidenced by reduced virus detection in OP swabs. This suggests that IL-18 may be playing a role in the host immune response, as observed for other infectious diseases^45^. Previous mouse studies have demonstrated that RAM and other MLV strategies induce limited virus replication and stimulate mucosal immunity. In this study focused on chickens, we concentrated exclusively on evaluating whether the vaccine elicited detectable serum HI responses indicative of protection, as these responses are more readily assessed in the field^31,40,41,46^.

Drinking water vaccination offers several advantages, including uniform and rapid delivery, reduced costs, and the potential for extensive mass vaccination, which is highly desirable for poultry species^47^. Several studies have demonstrated the suitability of drinking water vaccination with MLVs, which have been successfully used and established for other important poultry diseases such as avian metapneumovirus, Fowl pox, infectious laryngotracheitis, and infectious bronchitis^48,49^. Live recombinant Newcastle disease vectored vaccines expressing the hemagglutinin of H9N2 and H5N1 have also been administered via drinking water vaccination and protected chickens against homologous challenges^50,51^.

In this study, we investigated whether MLV-H9N2-IL would be immunogenic and protective in chickens when administered via drinking water using prime-only or prime-and-boost strategies. After mixing the vaccine in distilled water, we analyzed vaccine stability at different temperatures over time. MLV-H9N2-IL at 10^6^ EID_50_/ml remained stable during vaccine administration at 25 °C, 30 °C, and 40 °C. This is particularly important because temperatures inside chicken houses can easily reach 40 °C, which must be considered when delivering vaccines. MLV-H9N2-IL at 10^4^ EID_50_/ml was stable at 25 °C but exhibited a rapid decline in titers when incubated at 30 °C and 40 °C. Additionally, HI titers and neutralizing antibodies were readily detected after prime vaccination and increased after boost in a dose-dependent manner.

Both vaccine doses and strategies reduced viral loads after the homologous challenge in CL swabs, OP swabs, sinuses, trachea, and cloaca. Importantly, a prime-and-boost regimen at 10^6^ EID_50_/ml induced sterilizing immunity in chickens, as evidenced by a complete absence of virus replication in all tissues mentioned above. The ability of MLV-H9N2-IL to completely prevent virus replication and shedding after challenge is highly significant, as these birds would not transmit the virus to new hosts, effectively breaking the transmission cycle of avian influenza.

This study successfully demonstrates the generation and assessment of two MLVs using genome rearrangement. MLV-H9N2 and MLV-H9N2-IL are stable, grow similarly to an H9N2 WT virus in vitro, and exhibit an attenuated profile in vivo. Co-infection studies in vivo confirmed the lack of reassortment between MLV and WT virus segments. Additional studies beyond the scope of this report will be needed to demonstrate whether unmodified segments in the MLV can reassort and perpetuate through sustained transmission. Both vaccine candidates are immunogenic and protective in chickens when administered via the EROC route. MLV-H9N2-IL administered via drinking water is also immunogenic and protective in chickens, providing an alternative to individual bird vaccination. Overall, this work provides insights into the development of vaccines against avian influenza carrying molecular markers and immunomodulators and their potential for mass vaccination applications in the field.

The DIVA (Differentiating Infected from Vaccinated Animals) strategy uses vaccines that trigger a unique immune response compared to infection with the wild virus. However, most countries currently rely on traditional inactivated-adjuvanted vaccines for avian influenza, which do not inherently support DIVA. Only a few countries in Europe have recently initiated limited vaccination against highly pathogenic H5N1 viruses using recombinant vaccines and a DIVA approach^52^. Importantly, our focus is on a non-notifiable virus, H9N2 LPAIV, making DIVA less essential, especially where H9N2 is prevalent and poultry exports are not a major concern. Additionally, in less developed nations with limited diagnostic tools, the priority is outbreak control over distinguishing vaccinated and infected birds. It is also worth noting that the modified live virus (MLVs vaccines) created in this study contain one (HA) or two (HA and NA) instances of the Thosea asigna 2 A protease, which has an immunogenic C-terminal end^53^. Whether this holds true in chickens vaccinated with the rearranged MLVs requires further investigation. Future research will explore this and other MLV modifications that could enable DIVA implementation.

## Methods

### Cells and eggs

Madin-Darby canine kidney (MDCK) and human embryonic kidney 293 T cells (HEK293T) were a kind gift from Robert Webster (St Jude Children’s Research Hospital, Memphis, TN, USA). Human lung carcinoma (A549) cells were purchased from ATCC (CCL-185, Manassas, VA, USA). Cells were maintained in Dulbecco’s Modified Eagles Medium (DMEM, Sigma-Aldrich, St Louis, MO, USA) containing 10% fetal bovine serum (FBS, Sigma-Aldrich), 1% antibiotic/antimycotic (AB, Sigma-Aldrich) and 1% L-Glutamine (Sigma-Aldrich). Cells were cultured at 37 °C and 5% CO_2_. BCi-NS1.1 immortalized human airway epithelial (HAE) cells were a kind gift from Matthew S Walters (University of Oklahoma Health Sciences Center, Oklahoma City, OK, USA). Cells were maintained in PneumaCult-Ex Plus Basal Media (Stemcell, Vancouver, BC, Canada) containing 2% PneumaCult-Ex Plus 50X Supplement (Stemcell), 0.1% Hydrocortisone (Stemcell), 1% Penicillin/Streptomycin (Gibco, Billings, MT, USA), 0.5% Amphotericin B (Gibco), and 0.1% Gentamycin (Sigma-Aldrich) and cultured at 37 °C and 5% CO_2_. For differentiation of BCi-NS1.1 cells, the apical portion of 12-well ALI transwell plates (VWR, Radnor, PA, USA) were coated with 1X human collagen (Sigma-Aldrich) for 1 h. The 1X human collagen was then removed from the well, and the plates were incubated for 30 min. Then, the plates were seeded with 3 × 10^5^ HAE cells per well with basal media in the apical portion of the well and cultured at 37 °C and 8% CO_2_ until reaching 100% confluency. Pneumacult ALI Base Medium (ALI Media, Stemcell) was prepared with 10% 10X Pneumacult ALI Supplement (Stemcell), 1% Pen/Strep (Gibco), 0.5% Amphotericin B (Gibco), and 0.1% Gentamycin (Sigma-Aldrich). ALI-Complete media was prepared with 1% Pneumacult ALI Maintenance Supplement (Stemcell), 0.2% Heparin Solution (Stemcell), and 0.5% Hydrocortisone (Stemcell). After cells reached 100% confluency, the basal media was removed from the basal and apical portion of the wells. ALI-Complete media was added to the basal portion of the well. Cells were cultured at 37 °C and 8% CO_2_ for 5 days, and media was replenished every 2–3 days. After 5 days at 8% CO_2_, cells were cultured at 37 °C and 5% CO_2_ until the cells reached 28 days in ALI-Complete media. Media was replenished every 2–3 days. Specific pathogen-free embryonated chicken eggs (ECEs) used for virus propagation and stock titration were obtained from Charles Rivers (Wilmington, MA, USA).

### Generation of reverse genetics plasmids

The genome rearrangement strategies to produce MLVs have been previously described^40,42^. The reverse genetics backbone of the prototypical LPAIV strain A/guinea fowl/Hong Kong/WF10/1999 (H9N2) – G1/h9.4.1 lineage – has been previously described^29^. To generate the plasmid pDP-PB1M2WF10, the plasmid pDP-PB1WF10 was modified to preserve packaging signals in segment 2^54^ and carrying a chimeric PB1 ORF with a C-terminal tag consisting of a glycine-glycine-glycine-glycine-serine (G4S) spacer, the Thosea asigna virus 2 A protease (2a Tav) and the M2 ORF. The pDP-M1∆M2 was generated from the plasmid pDP-MWF10 by site-directed mutagenesis to carry multiple early stop codons in the M2 ORF to prevent its expression. The pDP-DRPAVIAN-HA-ck/EGY carries a chimeric HA segment derived from the H9N2 strain A/chicken/Egypt/A15068/2018 (ck/EGY) carrying a 58 amino acids long sequence, including the unique 8 amino acid peptide sequence aspartic acid-arginine-proline-alanine-valine-isoleucine-alanine-asparragine (DRPAVIAN) placed downstream of the HA signal peptide sequence but upstream of the mature HA ORF. To generate the plasmid pDP-NAckIL18, the plasmid pDP-NAWF10 was digested with BamHI and HindIII and then a synthetic fragment encoding the mature protein sequence of chicken IL-18 (ckIL18, Genscript, Piscataway, NJ) was subcloned at the C-terminus in frame with the NA ORF. Plasmids were propagated in TOP-10 *E. coli* cells (ThermoFisher, Waltham, MA, USA) and purified using the QIAGEN Plasmid Maxi Kit (Qiagen, Gaithersburg, MD, USA). Plasmid sequences were confirmed by Sanger Sequencing (Psomagen, Rockville, MD, USA).

### Generation of MLVs by reverse genetics

Recombinant viruses were rescued by reverse genetics using the 8-plasmid system and helper plasmids in co-cultured HEK293T and MDCK cells in 6-well plates^55^. The MLV-H9N2 virus was generated from the combination of reverse genetics plasmids pDP-PB2WF10, pDP-PB1M2WF10, pDP-PAWF10, pDP-DRPAVIAN-HA-ck/EGY, pDP-NPWF10, pDP-NAWF10, pDP-M1∆M2WF10, pDP-NSWF10 and the protein expression helper plasmids pcDNA774-PB1^56^ encoding the PB1 polymerase subunit from the laboratory adapted strain A/Puerto Rico/8/1934 (H1N1) (PR8) and pCAGGS-H1-HA from A/California/04/2009 (H1N1) (Ca/04)^33^. For the generation of MLV-H9N2-IL, the plasmid pDP-NAckIL18 was used instead of pDP-NAWF10 along with protein expression helper plasmids encoding the polymerase complex from PR8 (pcDNA762-PB2, pcDNA787-PA, pcDNA693-NP, pcDNA774-PB1)^56^, pCAGGS-H1-HA from Ca/04 and pCAGGS-N2-NA from A/turkey/Ohio/313053/04 (H3N2)^57^. On transfection day, 1 µg of each plasmid was mixed with the TransIT-LT1 transfection reagent (Mirus Bio LLC, Madison, WI, USA) in a ratio of 1 µg plasmid DNA/2 µL of transfection reagent in a final volume of 1 mL of Opti-MEM media (Fisher Scientific, Hampton, NH, USA). The mixture was incubated for 45 min and then used to overlay the 293 T/MDCK cells overnight. The next day, the transfection mixture was replaced with fresh Opti-MEM media containing 1% AB (Sigma-Aldrich). At 24 and 72 h post-transfection 1 µg/mL of tosylsulfonyl phenylalanyl chloromethyl ketone (TPCK) treated trypsin (Worthington Biochemicals, Lakewood, NJ, USA) was supplemented to the cells. Viral stocks were generated in 10-day-old specific pathogen-free (SPF) eggs. Allantoic fluids were harvested at 48 h post-infection (hpi), centrifuged, aliquoted, and stored at −80 °C. These stocks constitute the first passage in eggs (E1). Viruses were titrated by tissue culture infectious dose 50 (TCID_50_) and egg infectious dose 50 (EID_50_). Virus titers were established by the Reed and Muench method^58^. All viruses used in this study were confirmed by Sanger sequencing (Psomagen).

### Genome stability of MLVs

The MLVs were serially diluted 10-fold in phosphate-buffered saline (PBS) containing 1% AB (Sigma-Aldrich); 100 µL of 3 different dilutions were inoculated in 10-day-old SPF eggs. Eggs were incubated at 35 °C for 48 h, placed at 4 °C overnight and then allantoic fluid from each inoculated egg was collected to perform hemagglutination assays using 0,5% chicken red blood cells. For each MLV, the dilution with the highest HAU was harvested, centrifuged, aliquoted, and stored at −80 °C. Five serial passages were performed following the same procedure described above. The MLVs harvested from egg passage 5 (E5) were used to generate a viral stock. Viral RNA extraction of both MLVs (E1 and E5) was carried out using the QIAquick® Viral RNA Mini Kit (Qiagen, Germantown, MD, USA). Viral RNA was amplified by RT-PCR using SuperScript III One-Step RT-PCR Platinum Taq HiFi DNA polymerase (ThermoFisher). The PB1 segment was amplified using the forward primer PB1-2068F (5’-TTCTTGAGGATGAACAGATGTACCAGAAGTGC-3’) and reverse primer Bm-PB1-2341R-eq (5’-ATATCGTCTCGTATTAGTAGAAACAAGGCATTTTTTCATG-3’). The HA segment was amplified using the forward primer H9HA-1F (5’-CTAGCAGTTAACCGGAGTACTGG-3’) and the reverse primer H9HA657-693Rev (5’-GACAAGGGGCCTTGGCCCTATCACTGGTTTGAAGGTC-3’). The NA segment was amplified using the forward primer WF10NA118F (5’-GATTCGCGCTCTGGTTATGA-3’) and the reverse primer N2Hu_1463R (5’-AGTAGAAACAAGGAGTTTTTTC-3’). The amplicons were resolved on an agarose gel, purified using the QIAquick® Gel Extraction Kit (Qiagen), and analyzed by Sanger sequencing (Psomagen).

### Growth kinetics in vitro

Six-well plates were seeded with 3 × 10^5^ MDCK cells or 5 × 10^5^ A549 cells per well the day before virus inoculation. Once HAE cells reached 28 days in ALI-Complete media, cells were washed 3 times with PBS (Life Technologies) before inoculation. Then, MDCK, A549, and HAE cells were inoculated with either H9N2 WT, MLV-H9N2, MLV-H9N2-IL, H9N2att^29^, or pH1N1 [A/California/04/2009 (H1N1)]^33^ at a multiplicity of infection (MOI) of 0.01. Upon virus inoculation, cells were incubated for 15 min at 4 °C and then for 45 min at 37 °C or 41 °C. Subsequently, A549 and MDCK cells were washed twice with 1 ml of PBS (Life Technologies) and supplemented with 2 ml of Opti-MEM (Fisher Scientific) containing TPCK-trypsin (Worthington Biochemicals, Lakewood, NJ, USA) and 1% AB (Sigma-Aldrich) and further incubated at 37 °C or 41 °C and 5% CO_2_ for 96 h. HAE cells were washed twice with 1 ml of PBS (Life Technologies) and fresh ALI-Complete media was added to the basal portion of the well and further incubated at 37 °C or 41 °C and 5% CO_2_ for 96 h. Opti-MEM (Fisher Scientific) was added to the apical portion of the well, and the cells were incubated at 37 °C or 41 °C for 10 min before collecting the supernatant. For all cells, supernatants were collected at 0, 12, 24, 48, 72, and 96 hpi and used to quantify the levels of viral replication by real-time quantitative reverse transcriptase PCR (RT-qPCR). For this purpose, viral RNA (vRNA) was extracted using the MagMAXTM-96 viral RNA Isolation kit (ThermoFisher). The quantification of vRNA was based on the Influenza A matrix gene using the forward primer M + 25 (5’-AGATGAGTCTTCTAACCGAGGTCG-3’) and the reverse primer M-124 (5’-TGCAAAAACATCTTCAAGTCTCTG-3’) along with the qPCR probe M + 64 (5’-FAM-GGCCCCCTCAAAGCCGA-TAMRA-3’). Experiments were carried out in a Quantstudio 3 (Applied Biosystem, Foster City, CA, USA) using qScript XLT One-Step RT-qPCR ToughMix Quantabio (ThermoFisher). Standard curves to correlate quantitative PCR crossing-point values with virus titers were made using a 10-fold dilution from a virus stock of known titer. Virus titers after RT-qPCR were expressed as log_10_ TCID_50_ equivalents/ml (log_10_ TCID_50_ equivalent/ml).

### Western blotting

Confluent MDCK cells were inoculated with the MLVs (MLV-H9N2 and MLV-H9N2-IL) at an MOI of 1 or 10 for 1 h at 37 °C. Following inoculation, the inoculum was removed and replaced with fresh Opti-MEM (Fisher Scientific). Infected cells were incubated at 37 °C for 10 h. Moreover, confluent HEK293T cells were transfected with 10 µg of plasmid pCAGGS expressing N2WF10-Furin-2A-IL-18-HIS for 48 h at 37 °C. After incubation, the media was removed from the cells and cold RIPA buffer (ThermoFisher) was used to lyse the cells. Quantification of total proteins was performed using BCA protein assay kit (ThermoFisher). Then, 150 µl of supernatant was mixed with 150 µl of Laemmli buffer containing mercaptoethanol (Bio-Rad, Hercules, CA, USA), followed by boiling for 7 min, and brief sonication. Proteins were separated on a 12% sodium dodecyl sulfate-polyacrylamide gel electrophoresis (SDS-PAGE) gel and were transferred to nitrocellulose membranes (Bio-Rad) for immunoblot analysis. Membranes were blocked in 5% molecular-grade nonfat dry milk overnight on shaker at 4 °C. Then, membranes were incubated with a mouse primary antibody against chicken IL-18 (catalog number #EUS040, 1:500, Kerafast, Boston, MA, USA) or a mouse primary antibody against glyceraldehyde-3-phosphate dehydrogenase (GAPDH) (catalog number #AM4300, 1:500, Fisher Scientific) for 2 h shaking at room temperature following manufacturers’ recommendations. After washing, the membranes were incubated with the respective secondary antibodies diluted in 5% molecular-grade nonfat dry milk: goat anti-mouse secondary antibody (catalog number #31430, 1:5000, Fisher Scientific) and anti-mouse IgG-horseradish peroxidase (HRP) conjugate (catalog number #1610380, 1:5000, Fisher Scientific) for 1 h shaking at room temperature following the manufacturer’s recommendation. Finally, the membranes were washed and imaged through a chemiluminescent reaction using West Femto (ThermoFisher Scientific). The ChemiDoc MP Imaging System (Bio-Rad) was employed to visualize the membranes. The captured images in TIFF format are shown in Supplementary Fig. 3.

### Co-infection studies in vitro

MDCK cells were seeded in 6-well plates at a ratio of 3 × 10^5^ cells per well. The next day, cells were co-infected with a mixture of either MLV-H9N2 or MLV-H9N2-IL and A/Chicken/Tunisia/812/2012 (H9N2) (ck/Tun) at the ratio 10MLV:1WT as follows: MLV-H9N2 (MOI 0.01) + ck/Tun (MOI 0.001) and MLV-H9N2-IL (MOI 0.01) + ck/Tun (MOI 0.001). The co-infection was performed with 500 µl of each mixture for 15 min at 4 °C and then 45 min at 37 °C. Subsequently, the inoculum was removed, and cells were washed twice with 1 ml of PBS (Life Technologies). Finally, 2 ml of Opti-MEM (Fisher Scientific) supplemented with TPCK-trypsin (Worthington Biochemicals, Lakewood, NJ, USA) and 1% AB (Sigma-Aldrich, St. Louis, MO, USA) was added to the cells. Cells were incubated at 37 °C and 5% CO_2._ Supernatants collected at 72 hpi were used for limiting dilution assays as described elsewhere^16^ to isolate cloned populations. Briefly, a 96-well plate was seeded with 1.5 × 10^4^ MDCK cells/well. Cells were infected with 8, 10-fold serial dilutions of each virus combination. After 72 h at 37 °C, supernatants were collected from wells infected with the most diluted sample displaying cytopathic effect (CPE) and then used for the second round of infection by limiting dilution. Supernatants from the second round of infection were collected at 72 h and used for sequencing.

### Next-Generation Sequencing (NGS)

The vRNA from each sample was extracted using the MagMAX-96 viral RNA Isolation kit (ThermoFisher). Multi-Segment RT-PCR (MS-RTPCR) was performed as previously described^59^. Briefly, 2.5 µl of vRNA was used as a template in a 25 µl MS-RTPCR reaction (SuperScript III One-Step RT-PCR Platinum Taq HiFi DNA polymerase (ThermoFisher)) using the following primers: Opti1‐F1 5′‐GTTACGCGCCAGCAAAAGCAGG‐3′; Opti1‐F2 5′‐GTTACGCGCCAGCGAAAGCAGG‐3′; Opti1‐R1 5′‐GTTACGCGCCAGTAGAAACAAGG‐3′. The final product was analyzed in 1% agarose gel to verify whole genome amplification^60^. MS-RTPCR amplicons were sequenced using the Illumina platform as described elsewhere^61^. Briefly, products were purified using 0.45X of Agencourt AMPure XP Magnetic Beads (Beckman Coulter, Brea, CA, USA) and eluted in HyClone molecular biology water (Genesee Scientific, San Diego, CA, USA). The concentration of the samples was determined using a Qubit buffer kit (ThermoFisher, Waltham, MA, USA) in a Qubit 3.0 fluorometer (ThermoFisher) and normalized to 0.2 ng/µl. Indexes were added via tagmentation using the Nextera XT DNA library preparation kit (Illumina, San Diego, CA, USA). The reaction was set as 40% of the suggested final volume. Next, samples were purified using 0.7X of Agencourt AMPure XP Magnetic Beads and analyzed on a Bioanalyzer using a High Sensitivity DNA kit (Agilent, Santa Clara, CA, USA). Libraries were pooled and normalized to 0.5 nM. After denaturation, the final loading concentration of the pooled libraries was 14 pM. Libraries were sequenced using a MiSeq Reagent Kit V2, 300 cycles (Illumina, San Diego, CA, USA). Genome assembly was performed using a pipeline previously described^62^. For co-infection studies, FASTQ files generated were used to map the proportion (%) of NGS reads that matched either the MLV- or WT-specific segment. For unmodified segments (PB2, PA, NP, NS), the FASTQ file was used to map the reads to the whole WT or MLV segment, and the proportion was established based on the number of reads for the specific segment (WT or MLV) divided by the total number of reads for the given sample. To distinguish between WT or MLV-modified segments (PB1, HA, NA, and M), NGS reads were mapped to the gene segment junctions (PB1-M2; DRP-HA; NA-ckIL-18; M2esc).

### Preparation of WIV-adj vaccine control

Reverse genetics was used to prepare a virus carrying the H9 HA of ck/EGY and the NA of the WF10 virus in the background of the laboratory-adapted strain A/Puerto Rico/08/34 (H1N1). Upon virus rescue, stocks were grown in ECE. To inactivate the virus, allantoic fluid containing the live virus was mixed with Binary Ethyleneimine 0.1 M solution and incubated at 37 °C in a T175 flask (ThermoFisher). After 13 h, this solution was added to a new T175 flask and incubated for 7 h at 37 °C. Next, 1 M sodium thiosulfate solution was added to the BEI-treated virus stock, mixed thoroughly and the pH was adjusted to 7.2. An aliquot 1:10 (500 µl out of 5 mL) of the inactivated virus was inoculated into ECE and MDCK cells to confirm the inactivation process. On the day of vaccination, the vaccine emulsions were prepared by mixing Montanide ISA71 VG (Seppic, Fairfield, NJ, USA) with the inactivated virus at a 70:30 ratio Montanide:Virus at 512 HAU per dose.

### Vaccine-challenge studies in chickens

Studies were approved by the Institutional Animal Care and Use Committee (IACUC) at the University of Georgia (Animal Use Protocol A2022 09-001-Y1-A5) to be performed under ABSL2 conditions. SPF White Leghorn embryonated eggs (Charles River Laboratories International Inc., Wilmington, MA, USA) were hatched and chicks raised for 2 weeks. In the first experiment, 4 groups of 2-week-old chickens were established and inoculated through the eyes and the respiratory (nasal and tracheal), oral, and cloacal routes (EROC) with either 1 × 10^6^ EID_50_/chicken of MLV-H9N2 (*n* = 30), MLV-H9N2-IL (*n* = 30) or control PBS (mock vaccinated group; *n* = 35) or via the subcutaneous route with 512 HAU/chicken of the WIVadj-H9N2 vaccine (*n* = 30). Chickens were boosted 2 weeks after prime with the corresponding homologous vaccine using the same routes. Two weeks after the boost, chickens were challenged with 1 × 10^8^ EID_50_/chicken of the homologous virus via EROC route. The challenge virus was a recombinant virus encoding the wild-type H9 HA of ck/EGY in the background of the wild-type WF10 strain (ck/EGY/WF10). In the second experiment, 2-week-old SPF White Leghorn chickens were vaccinated via drinking water with the MLV-H9N2-IL vaccine at either the low dose of 10^4^ EID_50_/ml (*n* = 30) or the high dose of 10^6^ EID_50_/ml (*n* = 30) in a prime-only or prime and boost 2 weeks apart. A subset of chickens (*n* = 30) was administered water only. The MLV-H9N2-IL virus vaccine was diluted in distilled sterile water with Vac-Pac Plus (Best Veterinary Solutions, Columbus, GA, USA) to neutralize residual chlorine or salts in the water and stabilize the pH. Before vaccine administration, chickens were deprived of water for 6 h. Drinking water vaccination proceeded for 2 h with the MLV-H9N2-IL virus vaccine administered using bell drinkers. Two weeks after prime, half of the chickens (*n* = 15) in each group were boosted with the same doses as the prime. Two weeks after the boost or four weeks after prime, chickens were challenged with 1 × 10^8^ EID_50_/chicken of the ck/EGY/WF10 virus via EROC route as described previously.

### Transmission of MLV-H9N2 and MLV-H9N2-IL viruses and reassortment in vivo in chickens

Chickens (2-week-old) were inoculated with 1 × 10^6^ EID_50_/chicken via the EROC route with either the MLV-H9N2 (*n* = 4) or MLV-H9N2-IL (*n* = 4). At 24 hpi, directly inoculated chickens were moved into a clean isolator that contained naïve direct contact chickens (*n* = 4) for each respective group. Oropharyngeal (OP) and cloacal (CL) swabs were collected every day until 6 days post-inoculation (dpi) and 6 days post-contact. Viral loads were analyzed by RT-qPCR as described previously. In the fourth experiment, 2-week-old chickens were coinfected with the MLV-H9N2 (*n* = 4) or MLV-H9N2-IL (*n* = 4) at 1 × 10^6^ EID_50_/chicken and Ck/Tun wild-type virus at 1 × 10^5^ EID_50_/chicken (ratio 10MLV:1WT). Oropharyngeal (OP) and cloacal (CL) swabs were collected every day until 5 days days-post infection (dpi) and directly sequenced by NGS as described previously in this manuscript.

### Vaccine stability in drinking water

The MLV-H9N2-IL virus vaccine diluted in sterile water with Vac-Pac Plus was incubated at different temperatures in a thermocycler: 25 °C, 30 °C, 40 °C, and 50 °C. Timepoints were collected at 0, 1, 2, and 24 h after vaccine preparation. All timepoints were titrated by TCID_50,_ and vaccine stability expressed as the amount of infectious virus present at the specific timepoint with virus titers established by the Reed and Muench method^63^.

### Nucleoprotein enzyme-linked immunoassays

Blood was collected from a subset of chickens per group 12 days post-prime (dpp), and 12 days post-boost (dpb). The levels of nucleoprotein (NP) antibodies were determined using the IDEXX AI MultiS-Screen Avian Influenza Virus Antibody Test kit (IDEXX Laboratories Inc., Westbrook, ME, USA). The presence or absence of antibodies was determined by relating the absorbance values at 650 nm to the negative control mean. A threshold for positive samples of 0.6 was established following the manufacturer’s recommendations.

### Hemagglutination Inhibition (HI) assays

Blood was collected from a subset of chickens per group 12 dpp, and 12 dpb. In addition, blood was collected from a subset of chickens 26 dpp for the second experiment – drinking water vaccination – in the prime-only group. Sera samples were analyzed by hemagglutination inhibition assay as described elsewhere^41^ to detect the presence of neutralizing antibodies. Briefly, sera were treated twice with 50% chicken red blood cells (RBCs) and diluted 1:10 in PBS. Of this dilution, 50 μL were added to the first column of a 96-well plate and 2-fold serially diluted. Then, sera were mixed with 4 HAU/25 μL of the homologous virus. The virus/sera mixture was incubated for 30 min at room temperature and the HI activity was determined after 30 min of incubation with 0.5% of chicken RBCs. HI titers below 10 were arbitrarily assigned a value of 10.

### Virus neutralization assays

Blood was collected from a subset of chickens per group 12 dpp, and 12 dpb. In addition, blood was collected from a subset of chickens 26 dpp for the second experiment – drinking water vaccination – in the prime-only group. Serum samples were treated twice with 50% RBCs as described previously. Serum samples were used to perform virus neutralization assays as described elsewhere^41^ but modified to rely on Nanoluciferase activity (VNLuc)^64^. Briefly, we generated a homologous H9N1 influenza virus in the PR8 backbone carrying a Nanoluciferase gene downstream of the PB1 gene^64^. Sera samples were serially diluted in a 96-well plate and incubated with 100 TCID_50_ of the homologous virus. The serum/virus mixture was incubated for 1 h at 37 °C and then overlayed on MDCK cells for 15 min at 4 °C and 45 min at 37 °C. The mixture was removed, cells were supplemented with Opti-MEM-AB and TPCK and incubated for 48 h as described previously. VNluc titers were read out using the Nano-Glo Luciferase Assay System (Promega, Madison, WI, USA) with a Victor X3 multilabel plate reader (PerkinElmer, Waltham, MA, USA).

### Virus titration

OP and CL swabs were collected on 3 and 5 dpp to determine MLV and/or WT virus replication in the intestinal and respiratory tracts. In addition, OP and CL swabs were collected at 1-, 3-, 5-, and 7-days post-challenge (dpc). Briefly, swab samples were spun down, 10-fold serially diluted, and directly inoculated into a 96-well plate seeded with 1.5 × 10^4^ MDCK cells/well. Sinuses, trachea, lungs, pancreas, and cloaca were collected on days 3 post-challenge to analyze vaccine protection after homologous challenge. Tissue homogenates were generated using the Tissue Lyzer II (Qiagen, Hilden, Germany). Briefly, 1 mL of PBS-AB was added to each sample with Tungsten carbide 3 mm beads (Qiagen, Hilden, Germany) in the tubes. Samples were homogenized for 10 min and then centrifuged at 15,000 *g* for 10 min Supernatants were collected, aliquoted, and directly inoculated into a 96-well plate seeded with 1.5 × 10^4^ MDCK cells/well. All samples were titrated by TCID_50_ and virus titers were established by the Reed and Muench method^63^. Alternatively, samples were also titrated by RT-qPCR. For this purpose, vRNA was extracted using the MagMAXTM-96 viral RNA Isolation kit (ThermoFisher) and the quantification of vRNA was based on the Influenza A matrix gene as previously described in the current manuscript.

### Graphs/Statistical analyses

All data analyses and graphs were performed using GraphPad Prism software version 10 (GraphPad Software Inc., San Diego, CA, USA). Ordinary one-way or two-way ANOVA as needed were performed to calculate *P* values followed by Tukey’s multiple comparison tests. A *P* value below 0.05 was considered significant.

### Supplementary information

Supplementary Information

## Data Availability

All data generated, analyzed and included in this manuscript are available upon request.
