# Supplementary material for: Mass vaccination with reassortment-impaired live H9N2 avian influenza vaccine
Source: NPJ Vaccines. 2024 Aug 3;9:136. doi: 10.1038/s41541-024-00923-y (PMC11297921; doi:10.1038/s41541-024-00923-y)
Supplement: Supplementary file 1 — Supplementary Information [file 41541_2024_923_MOESM1_ESM.pdf]

Mass Vaccination with Reassortment-Impaired Live H9N2 Avian Influenza Vaccine

Flavio Cargnin Faccin, et al.

Supplementary data

* Address correspondence to Daniel R. Perez, dperez1@uga.edu

MLV RAM backbone

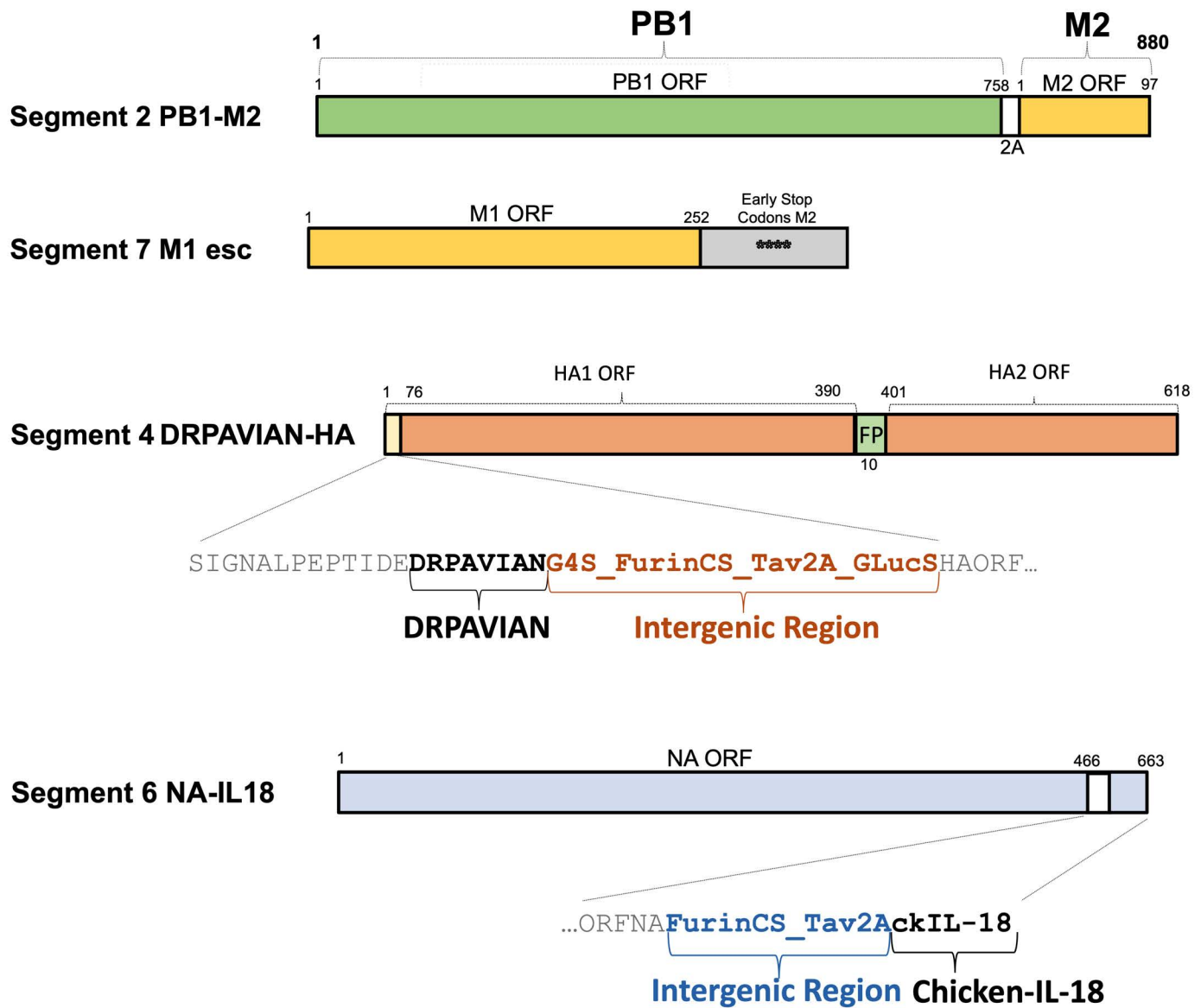

Supplementary Figure 1: Genome Modifications in MLV-H9N2 and MLV-H9N2-IL Vaccine Candidates.

Segment 2 Rearrangement (PB1-M2): The Reassortment Attenuation Method (RAM) strategy involves the relocation of the M2 open reading frame (ORF) to a position downstream of the PB1 ORF. This results in the creation of a chimeric PB1-M2 construct consisting of 880 amino acids, encoded within a 2640 nucleotide-long segment 2.

Segment 7 modification (M1ΔM2): To prevent the synthesis of the original M2 protein from segment 7, multiple stop codons were introduced into its ORF without altering the segment's length of 1027 nucleotides.

Segment 4 Modification (HA): Both the MLV-H9N2 and MLV-H9N2-IL candidates feature a modified segment 4. This segment encodes a chimeric hemagglutinin (HA) ORF comprising 618 amino acids within a 1854 nucleotide-long gene segment. A unique 58-amino acid sequence containing the DRPAVIAN peptide has been inserted downstream of the HA signal peptide but upstream of the mature HA ORF.

Segment 6 Modification (NA-IL18, MLV-H9N2-IL only): In the MLV-H9N2-IL candidate, the mature chicken interleukin 18 (ckIL18) protein sequence has been inserted in frame downstream of the C-terminus of the N2 neuraminidase (NA). A spacer sequence, cleaved during translation by 2ATav activity, separates the NA and ckIL18 sequences. This modification results in a 1989 nucleotide-long segment 6 encoding a chimeric NA-IL18 ORF of 663 amino acids.

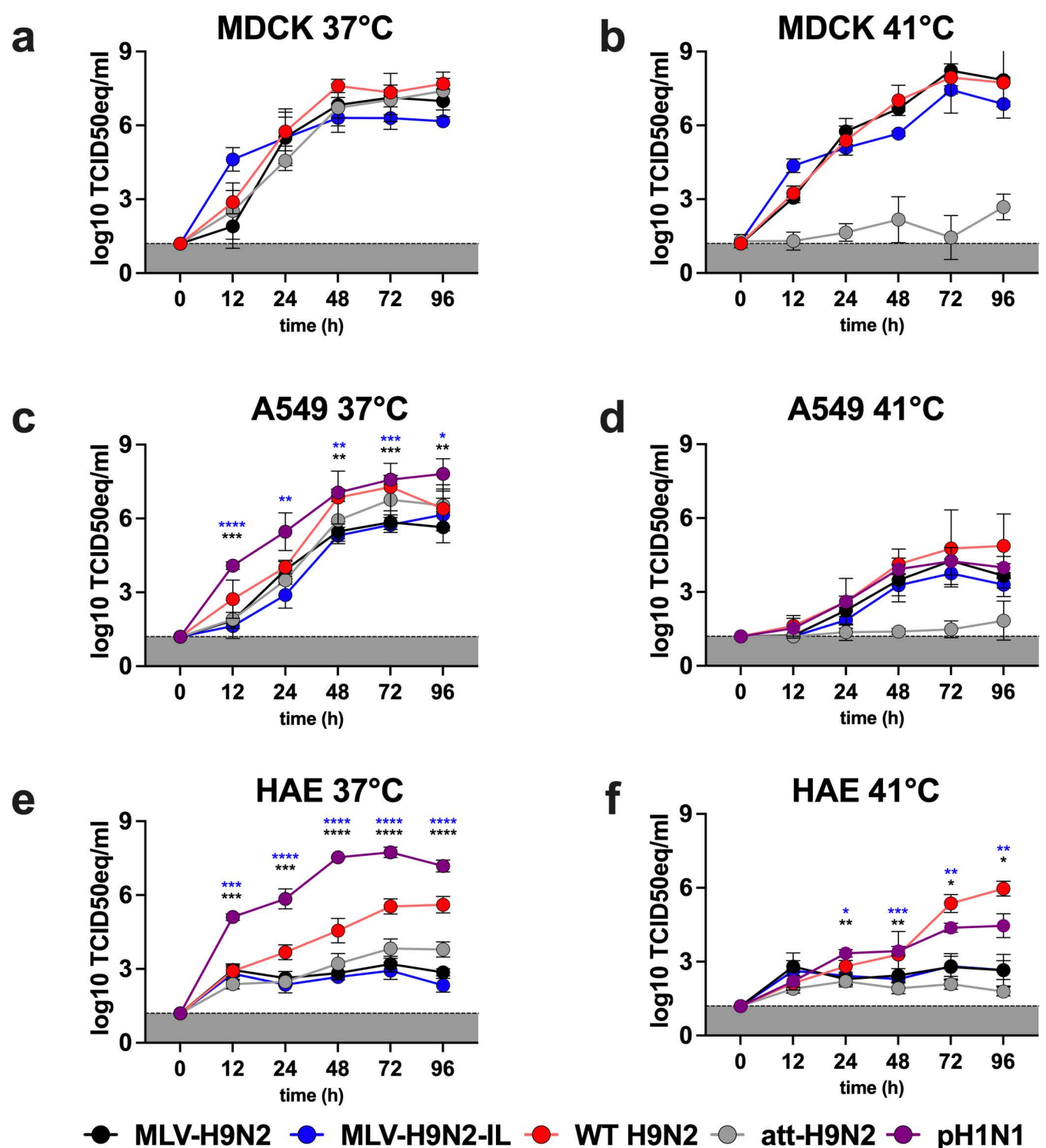

Supplementary Figure 2. The MLV-H9N2 and MLV-H9N2-IL exhibit stability in MDCK cells but replicate significantly less than a pandemic H1N1 virus in human-derived cells. Growth kinetics profiles of MLVs in MDCK cells at 37°C (a) and 41°C (b). RAM viruses grow at 41°C, unlike the att-H9N2 temperature-sensitive control. Growth kinetics were also performed in A549 cells at 37°C (c) and 41°C (d), and in HAE cells at 37°C (e) and 41°C (f). Overall, RAM viruses replicate significantly less than a pandemic H1N1 virus in human-derived cells. Cells were inoculated with either the MLV-H9N2 (black), MLV-H9N2-IL (blue), H9N2 WT (red), att-H9N2 (gray), or the pandemic H1N1 (purple), at an MOI of 0.01 and incubated at 37°C or 41°C for 96 h. Samples were collected at 0, 12, 24, 48, 72, and 96 hpi and titrated by RT-qPCR. Virus titers are shown as the mean $\pm$ SD Log₁₀ TCID₅₀ equivalents/mL of duplicates and two independent experiments. Samples with undetected virus titers were assigned the limit of detection value (LOD, 1.199 Log₁₀ TCID₅₀ equivalent/mL). Data analysis and graphs performed with Prism v10 (GraphPad) using ordinary two-way ANOVA, P values calculated by Tukey's multiple comparison tests. Significant differences between viruses are indicated by asterisks (*) as follows: * = $p < 0.05$, ** = $p < 0.005$, *** = $p < 0.0005$, and **** = $p < 0.0001$. Blue and black asterisks represent, respectively, significant statistical differences comparing the MLV-H9N2-IL and MLV-H9N2 to the pH1N1 virus.

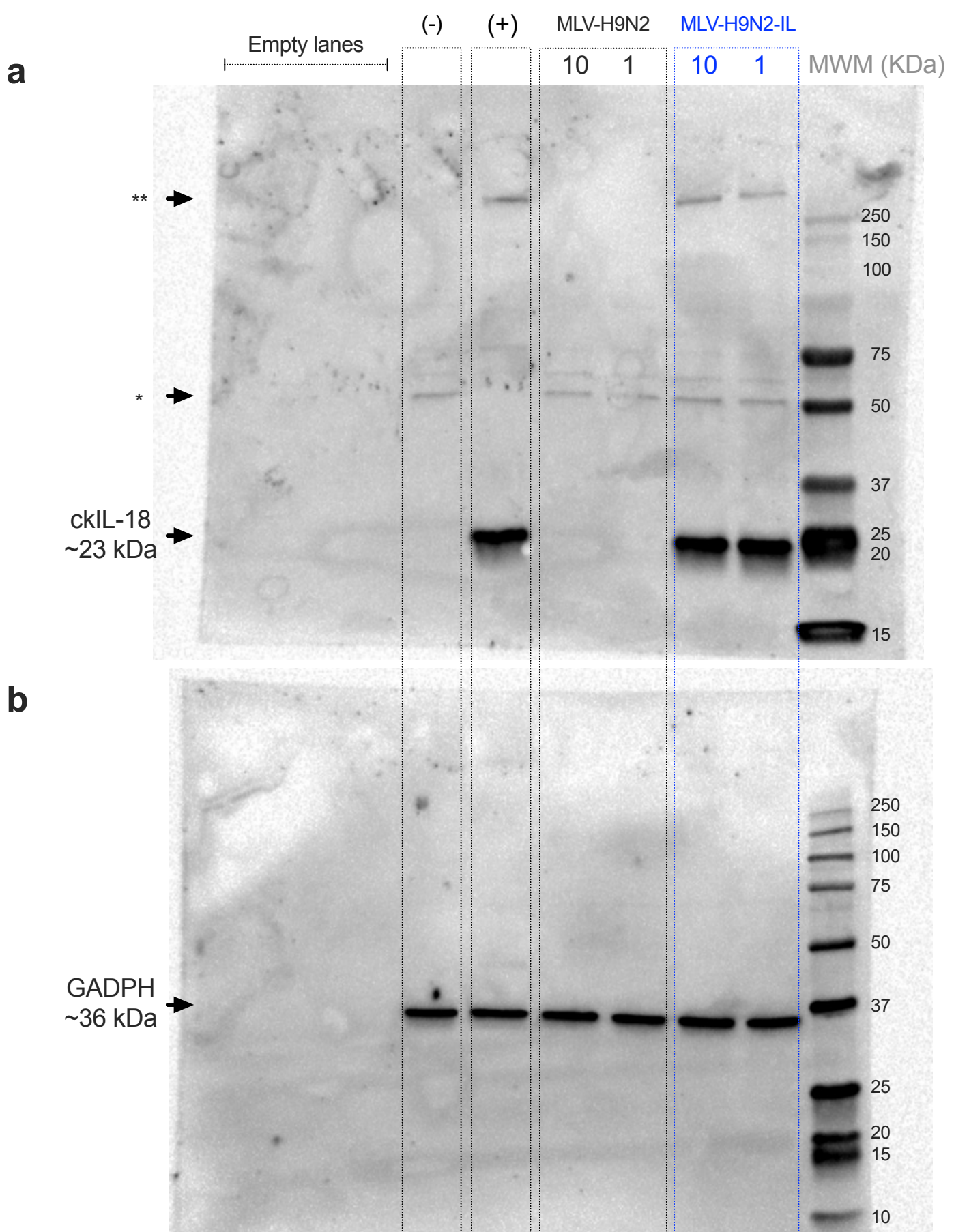

Supplementary Figure 3. Chicken IL-18 expression from the MLV-H9N2-IL virus by western blot. Protein lysates of MDCK cells inoculated with MLV-H9N2 (black) or MLV-H9N2-IL (blue) at an MOI of 10 or 1. Positive control corresponds to protein lysates of HEK293T cells transfected with the control expression plasmid pCAGGS expressing N2WF10-Furin-2A-IL-18-HIS. Negative control corresponds to non-transfected HEK293T cell lysates. The arrows indicate the predicted molecular weight of (a) chicken IL-18 (~23 KDa) and (b) the host cellular protein glyceraldehyde-3-phosphate dehydrogenase (GAPDH; ~36 KDa) used as a gel loading control. MWM is the molecular weight marker and sizes are indicated in the figure. *Non-specific bands are indicated in the gel. **Unknown protein bands present on samples that express ckIL-18.
